# Defining the diagnostic divide: an analysis of registered radiological equipment resources in a low-income African country

**DOI:** 10.11604/pamj.2016.25.99.9736

**Published:** 2016-10-20

**Authors:** Patrick Sitati Ngoya, Wilbroad Edward Muhogora, Richard Denys Pitcher

**Affiliations:** 1 Division of Radiodiagnosis, Department of Medical Imaging and Clinical Oncology, Faculty of Medicine and Health Sciences, Stellenbosch University and Tygerberg Academic Hospital, Cape Town, South Africa; 2 Directorate of Radiation Control, Tanzania Atomic Energy Commission, Arusha, Tanzania

**Keywords:** Tanzania, low-income country, diagnostic radiology, imaging equipment resources

## Abstract

**Introduction:**

Diagnostic radiology is recognised as a key component of modern healthcare. However there is marked inequality in global access to imaging. Rural populations of low- and middle-income countries (LMICs) have the greatest need. Carefully coordinated healthcare planning is required to meet the ever increasing global demand for imaging and to ensure equitable access to services. However, meaningful planning requires robust data. Currently, there are no comprehensive published data on radiological equipment resources in low-income countries. The aim of this study was to conduct the first detailed analysis of registered diagnostic radiology equipment resources in a low-income African country and compare findings with recently published South African data.

**Methods:**

The study was conducted in Tanzania in September 2014, in collaboration with the Tanzanian Atomic Energy Commission (TAEC), which maintains a comprehensive database of the country’s registered diagnostic imaging equipment. All TAEC equipment data were quantified as units per million people by imaging modality, geographical zone and healthcare sector.

**Results:**

There are 5.7 general radiography units per million people in the public sector with a relatively homogeneous geographical distribution. When compared with the South African public sector, Tanzanian resources are 3-, 21- and 6-times lower in general radiography, computed tomography and magnetic resonance imaging, respectively.

**Conclusion:**

The homogeneous Tanzanian distribution of basic public-sector radiological services reflects central government’s commitment to equitable distribution of essential resources. However, the 5.7 general radiography units per million people is lower than the 20 units per million people recommended by the World Health Organization.

## Introduction

A number of healthcare imperatives are being brought to bear on diagnostic imaging. These are contributing to challenging discourses in the domain and are likely to impact the future of global radiological practice. The past half-century has seen a series of important technological advances in diagnostic imaging, including the introduction of ultrasound, computed tomography (CT), magnetic resonance (MR), functional imaging and picture archiving and communication systems (PACS). These advances have increased the clinical use of radiological services, enhanced the value of radiology to individual patients and bolstered the overall sustainability of healthcare systems [1–3]. Diagnostic imaging is now recognised as a key component of comprehensive healthcare, through its contributions to preventive health programs, definitive diagnostic work-up and assessment of treatment response [4, 5]. Furthermore, basic radiological services are now deemed mandatory for the effective provision of primary care [6–9].

Between 1988 and 2008, the number of diagnostic imaging studies performed globally more than doubled [10]. This demonstrates the relentless increase in the global demand for radiological services, which has been driven by technical advances in imaging, together with global population growth, longer life expectancy, a rise in chronic diseases and the HIV pandemic [11, 12]. The expanding global demand for imaging represents an important challenge for modern healthcare, since radiological services are capital- and labor-intensive, particularly for the more sophisticated modalities [5, 8, 13, 14]. It has been estimated that imaging currently contributes 10% to the total per capita healthcare expenditure [15]. The present demand for diagnostic imaging exceeds global service capacity, although this analysis is complicated by stark inequalities in worldwide access to imaging [16–18]. At one end of the radiological continuum are high-income countries with an abundance of sophisticated radiological resources, where there are concerns of over-utilization of imaging services and questions around the sustainability of imaging practices [19–24]. At the other end of the spectrum are the estimated one-half to two-thirds of the world’s population who lack access to basic medical imaging. The need is greatest amongst the rural populations in low- and middle-income countries. This has been termed the “radiology divide” [7, 13, 18, 25–27].

As a society, we need to reflect on our commitment to equitable access to good-quality health services and to entrench access to healthcare as an essential human right, realising that investment in health systems will promote societal cohesion and economic productivity [28, 29]. The principle of distributive justice is thus increasingly being embraced to address global inequalities in healthcare, including access to diagnostic imaging [30]. To this end, a number of ambitious projects have been initiated in the radiological domain, underpinned by the realization that an estimated ninety percent of all imaging needs in low- and middle-income countries (LMICs) can potentially be addressed by the provision of one simple x-ray unit and a basic ultrasound machine for every 50,000 people [6, 18, 27]. However, concern has been expressed around the long-term sustainability of random philanthropic or donor initiatives undertaken without comprehensive needs assessments or appropriate medium- to long-term planning [13].

There is a growing appreciation of the need for careful, coordinated strategic healthcare planning at national and international level, to meet burgeoning global service demands and ensure equitable access to care, particularly in the current economic crisis, which is expected to have far-reaching healthcare ramifications for all countries, regardless of economic status [31, 32]. There are also increasing pressures to ensure responsible utilization of radiological resources [33]. Although there is a plethora of documentation outlining appropriate use of imaging services in well-resourced environments, there has been no work on the impact of lack of radiological resources on clinical outcomes and no attempt to define an absolute minimum requirement for imaging in the achievement of health for all. The limited work to date suggests that lack of radiological resources contributes to inappropriate use of existing resources [20, 34]. Furthermore, there has been only limited work on assessing a population’s overall access to imaging [35].

Meaningful planning however requires robust data. In the radiological domain there are surprisingly limited published data on installed diagnostic imaging equipment resources at national level, especially in LMICs[36, 37]. Although the World Health Organization (WHO) has published national estimates of high-end medical imaging resources based on questionnaire surveys of member countries, these data do not include basic equipment such as general radiography units [38]. The limited available WHO data document marked disparities in the high-end resources amongst countries in the same World Bank economic class. The only comprehensive, country-based analysis of registered radiological equipment published to date is from South Africa, a middle-income country, where striking disparities have been documented both between the public and private healthcare sectors as well as geographical regions within the public sector [36]. However, the determinants of a nation’s radiological resources remain poorly understood and inadequately researched. Furthermore, associations between a country’s World Bank economic class, healthcare expenditure and diagnostic imaging services have not been critically evaluated. There has also been no comprehensive analysis of the registered diagnostic imaging equipment of low-income countries.

It is in this context that the Division of Radiodiagnosis of the Department of Medical Imaging and Clinical Oncology at Stellenbosch University has embarked on a systematic evaluation of the diagnostic radiology resources of African countries, with a view to providing baseline data that would contribute to an enhanced understanding of radiological services, and facilitate healthcare planning in LMICs.

The aim of this study was therefore to conduct a detailed analysis of registered diagnostic radiology equipment resources in a low-income African country, and to compare findings with recently published South African data.

## Methods

The study was conducted in the United Republic of Tanzania, a low-income African country, with a land area of 886,000 square kilometres (km2), comprising Tanzania Mainland and Zanzibar [39]. Tanzania has a total population of 44.9 million people according to the 2012 National Census, with the population being predominantly rural (70%) and with at least 16% of the population having health insurance [39, 40] The country has a gross domestic product (GDP) of approximately 50 billion US dollars and spends an estimated 3.5 billion USD (7% of GDP) on healthcare [41, 42].

The study was conducted in September 2014, in collaboration with the Directorate of Radiation Control of the Tanzanian Atomic Energy Commission (TAEC), which maintains a comprehensive database of the country’s registered diagnostic radiology equipment. All equipment data were as quantified as units per million people by imaging modality, geographical zone and healthcare sector. Findings were compared to recently published South Africa data [36].

General radiography (GR), fluoroscopy (FL), mammography (MM), computed tomography (CT) and magnetic resonance (MR) modalities were included in the analysis. Ultrasound (US) was excluded, as units are not registered by TAEC. For the purpose of this analysis, Tanzanian regions were combined into six geographical zones namely; Central (Dodoma, Singida, Tabora), Coastal (Dar es Salaam, Lindi, Morogoro, Mtwara, Pwani), Lake (Geita, Kagera, Kigoma, Mara, Mwanza, Shinyanga, Simiyu), Northern (Arusha, Manyara, Tanga, Kilimanjaro), Southern (Iringa, Katavi, Mbeya, Njombe, Rukwa, Ruvuma) and Zanzibar.

The study was approved by the TAEC and the Health Research Ethics Committee of the Faculty of Medicine and Health Sciences of the Stellenbosch University, Cape Town, South Africa (S14/07/152).

## Results

Tanzania’s diagnostic imaging equipment resources are reflected in Table 1.

**Table 1:** Tanzania's diagnostic radiology equipment units per million population by geographical zone and by health sector

Zone (Mil. Pop.+)	Population Density++	General Radiography			Fluoroscopy			Mammography			CT			MR		
		Public	Private	Total	Public	Private	Total	Public	Private	Total	Public	Private	Total	Public	Private	Total
Central (5.7)	0.34	5.26	17.97	7.37	0.84	0	0.70	0.21	0	0.18	0	0	0	0	0	0
Coastal (9.8)	0.52	7.59	52.25	15.00	1.10	6.15	1.94	0.49	3.07	0.92	0.12	5.53	1.02	0.24	1.23	0.41
Lake (14.0)	0.89	3.34	9.47	4.36	0.51	0	0.43	0.09	0	0.07	0.09	0.86	0.21	0	0	0
Northern (6.8)	0.56	7.58	33.66	11.91	1.06	0.89	1.03	0.35	0	0.29	0.18	3.54	0.74	0	0	0
Southern (7.3)	0.29	5.75	21.46	8.36	0.82	2.48	1.10	0.16	0	0.14	0	0.83	0.14	0	0	0
Zanzibar (1.3)	5.20	7.38	23.17	10.00	0.92	0	0.77	0	0	0	0	0	0	0	0	0
**Total (44.9)**	**0.51**	**5.66**	**25.89**	**9.02**	**0.83**	**1.88**	**1.00**	**0.24**	**0.67**	**0.31**	**0.08**	**2.15**	**0.42**	**0.05**	**0.27**	**0.09**
**Public : Private**			**1:5**			**1:2**			**1:3**			**1:27**			**1:5**	

+ Million Population

++ Million population per 10,000km^2^ of land area

### Public sector

The findings demonstrate an intuitive, price-driven hierarchy of access to imaging, with modality availability inversely related to relative unit cost. Thus, the least expensive modality is most available, and modalities become progressively less available with increasing cost. General radiography and fluoroscopy are available in almost all geographical zones and have a relatively homogeneous distribution. The overall level of resourcing in the public sector is low, with only 5.7 general radiography units per million people, which is well short of the WHO recommendation of 20 units per million people [6, 18, 36]. There is limited access to CT and MR units in the public sector, as demonstrated by CT being available in only 3 out of the 6 zones and MR in 1 out of the 6 zones. The overall high ratio of 70 general radiography units to 1 CT unit in the public sector underscores the preferential access to basic imaging services. CT access is seemingly influenced by population density and urban location, with the most densely populated urban zones having better availability. The Coastal zone, in particular the Dar es Salaam region, emerges as the country’s main referral centre, being the only region with the full spectrum of modern imaging modalities. Although the Lake zone has the largest population, it is the most poorly resourced, with the lowest number of equipment units per million people across all modalities.

### Private sector

The cost-driven hierarchy of access to imaging evident in the public sector is not clearly replicated in the private sector. The distribution of general radiography units in the private sector is less homogeneous than in the public sector, with a 5-fold discrepancy between the least and best resourced regions. The most poorly resourced private sector zone has more general radiography units than its best-resourced public sector counterpart. However, the overall level of private resources, at 25 general radiography units per million people is above the 20 units recommended by the WHO [6, 18, 36]. The relative accessibility of more sophisticated imaging services in the private sector is underscored by there being 1 CT for every 10 general radiography units.

### Public sector radiological resources: Tanzania vs. South Africa

A comparison of the Tanzanian and South African public sector radiological resources is presented in Table 2. As a middle-income country, South Africa spends 12 times more on public healthcare than Tanzania, a low-income country [36, 41, 42]. South Africa public-sector radiological resources show the same intuitive price-driven hierarchy of access to imaging as those in Tanzania. However, South Africa has greater resources across all modalities, with 3-, 21- and 6-times more resources in general radiography, CT and MR respectively, compared to Tanzania. South Africa (19.6 units per million people) approximates the minimum recommended WHO standard of 20 general radiography units per million people [6, 18, 36].

**Table 2:** Public sector radiological resources: Tanzania vs South Africa

A. Demographics:	Tanzania	South Africa
Million population (annual growth rate)	44.9 (2.6%)	54.0 (1.58%)
Area (1000km^2^)	890.1	1,219.1
GDP in billion US$ (annual growth)	48.06 (7.0%)	350.14 (1.5%)
Health expenditure per capita in US$	49	593
Health Insurance (% of the total population insured)	Yes (16.6%)	Yes (17%)
**B. Public Sector Diagnostic Radiology Equipment in Units per Million Population:**	**Tanzania**	**South Africa**
General Radiography	5.66	19.8
Fluoroscopy	0.83	2.5
Mammography	0.24	1.29
CT	0.08	1.7
MR	0.05	0.3

## Discussion

This is the first comprehensive quantitative analysis of registered diagnostic radiology equipment resources in a low-income African country. It thus provides useful baseline data for healthcare planning at national level, but also contributes to understanding regional healthcare challenges in Africa. Furthermore, this is the first comparative study detailing differences in radiological resources between a low-income and a middle-income African country, thereby contributing to the discourse on the minimum level of radiological equipment required to render effective care in resource-constrained environments.

The finding that Tanzania’s basic radiological equipment resources within the public sector are relatively homogeneously distributed across geographic zones reflects effective central government control of healthcare services, as well as a commitment to equitable distribution of essential resources. The 5.7 general radiography units per million people in the Tanzanian public sector is lower than the 20 units per million people recommended by the WHO [6, 18]. This defines the approximate shortfall in basic radiological services and informs strategic healthcare planning going forward. Furthermore, the country’s defined deficit in general radiography units could serve as a proxy estimate of the additional radiological human resources required to coordinate a future national general radiography service.

Tanzanian health services are based on a pyramidal referral pattern. The most basic care is the home-based preventive service provided by village health workers. Upward referral is then through ward-based dispensary services caring for up to 10,000 people, to health centres responsible for an average of 50,000 people in a single administrative division. From health centres, patients sequentially access district, regional and consultant hospitals with progressively higher levels of resources and staff expertise [43]. If the WHO recommendation [6, 18] of one general radiography unit per 50,000 people is to be realized, Tanzania should consider equipping each health centre with at least one general radiography unit.

The WHO has estimated that 90% of all imaging requirements in resource-constrained environments can be provided by the basic modalities of general radiography and ultrasound [6, 18]. Conversely, approximately 10% of imaging in such settings will require more sophisticated investigations such as CT and MR. Although the optimal ratio of CT scanner to general radiography unit in resource-limited environments has not been defined, extrapolation of the WHO estimate suggests that approximately 1 CT scanner is required for every 10 general radiography units. The finding that Tanzania’s public sector has one CT scanner for every 70 general radiography units underscores the country’s radiological challenges, and highlights a planning dilemma. The conundrum is how best to address the shortfall in both basic and more sophisticated imaging modalities within existing economic constraints. This is likely a common challenge in sub-Saharan Africa, since 33 countries in the region have public healthcare budgets equivalent to, or less than, Tanzania [44]. As a general rule, the acquisition of basic imaging equipment should be accorded priority, with targets for general radiography units ideally achieved prior to embarking on the roll-out of more sophisticated imaging.

However, there is increasing recognition that diagnostic imaging requirements should not be seen in isolation, but rather evaluated in the broad context of healthcare imperatives. Radiological services must be accessible, affordable and appropriate, and be seamlessly integrated into the overall healthcare system, to meet public health needs as defined by the local burden of disease [45]. Much work is thus still required to define the minimum radiological service needs of individual low-income countries. Sound reasoning and a solid evidence base is required in defining such need.

The strength of this quantitative work is its foundation on the TAEC official database of registered diagnostic imaging equipment, together with the TAEC’s full collaboration in the project. A limitation is the absence of a qualitative component to assess equipment functionality. It is possible that this introduced an overall positive bias in Tanzania’s public sector equipment resources, since previous work by Sungita et al has highlighted challenges in maintenance and quality assurance of public sector diagnostic imaging equipment [46]. A further limitation is the failure to include diagnostic ultrasound equipment, which is not registered with the national regulatory authority, since it does not involve ionizing radiation. This limitation is common to all current analyses of national diagnostic imaging resources and is a major constraint in the evaluation of the imaging capacity in LMICs, where ultrasound has the potential to play a pivotal role. To facilitate healthcare planning, registration of all diagnostic ultrasound equipment would be prudent.

## Conclusion

The homogeneous Tanzanian distribution of basic public sector radiological services reflects central government’s commitment to equitable distribution of essential resources. However, the 5.7 general radiography units per million people in the public sector is lower than the 20 units per million people recommended by the WHO, defining the country ’s diagnostic divide.

### What is known about this topic

- Currently, there is limited data on national diagnostic radiology equipment resources in low-income African countries;
- The only comprehensive data on national diagnostic radiology equipment resources published to date emanates from South Africa, a middle-income African country.

### What this study adds

- This is study provides a comprehensive analysis of national diagnostic radiology equipment resources in a low-income African country;
- The defined national diagnostic radiology equipment deficits could serve as a proxy estimate of additional human resources required to provide a more comprehensive imaging service in low-income countries.
